# Predicting male fertility in dairy cattle using markers with large effect and functional annotation data

**DOI:** 10.1186/s12864-019-5644-y

**Published:** 2019-04-02

**Authors:** Juan Pablo Nani, Fernanda M. Rezende, Francisco Peñagaricano

**Affiliations:** 1 Department of Animal Sciences, University of Florida, 2250 Shealy Drive, Gainesville, FL 32611 USA; 2 Estación Experimental Agropecuaria Rafaela, Instituto Nacional de Tecnología Agropecuaria, 22-2300 Rafaela, SF Argentina; 3 Faculdade de Medicina Veterinária, Universidade Federal de Uberlândia, Uberlândia, MG 38410-337 Brazil; 4 University of Florida Genetics Institute, University of Florida, Gainesville, FL 32610 USA

**Keywords:** Biological informed models, Kernel-based prediction, Sire conception rate

## Abstract

**Background:**

Fertility is among the most important economic traits in dairy cattle. Genomic prediction for cow fertility has received much attention in the last decade, while bull fertility has been largely overlooked. The goal of this study was to assess genomic prediction of dairy bull fertility using markers with large effect and functional annotation data. Sire conception rate (SCR) was used as a measure of service sire fertility. Dataset consisted of 11.5 k U.S. Holstein bulls with SCR records and about 300 k single nucleotide polymorphism (SNP) markers. The analyses included the use of both single-kernel and multi-kernel predictive models fitting either all SNPs, markers with large effect, or markers with presumed functional roles, such as non-synonymous, synonymous, or non-coding regulatory variants.

**Results:**

The entire set of SNPs yielded predictive correlations of 0.340. Five markers located on chromosomes BTA8, BTA9, BTA13, BTA17, and BTA27 showed marked dominance effects. Interestingly, the inclusion of these five major markers as fixed effects in the predictive models increased predictive correlations to 0.403, representing an increase in accuracy of about 19% compared with the standard model. Single-kernel models fitting functional SNP classes outperformed their counterparts using random sets of SNPs, suggesting that the predictive power of these functional variants is driven in part by their biological roles. Multi-kernel models fitting all the functional SNP classes together with the five major markers exhibited predictive correlations around 0.405.

**Conclusions:**

The inclusion of markers with large effect markedly improved the prediction of dairy sire fertility. Functional variants exhibited higher predictive ability than random variants, but did not outperform the standard whole-genome approach. This research is the foundation for the development of novel strategies that could help the dairy industry make accurate genome-guided selection decisions on service sire fertility.

## Background

Suboptimal reproductive performance in dairy cattle, specifically in Holstein purebred, is still a concerning problem in today’s dairy industry. Reproduction inefficiency in dairy cattle has a direct impact on the overall herd profitability by leading the system to reduced incomes (longer calving intervals, reduced milk yield, higher culling rates) and additional expenditures (cost of artificial insemination technician, frequent veterinary visits, hormone treatment, diagnosis and treatment costs) [1, 2].

Causes of poor fertility in dairy cows have been well studied [3] and efforts have been made to improve fertility traits by selective breeding. Three female fertility traits are routinely evaluated in the US national genetic evaluation with considerable success, along with health and other low heritability traits [4]. On the other hand, the genetic improvement of bull fertility has been largely ignored. This appears to be contradictory considering that semen from one service sire bull is used to inseminate hundreds of cows and, thus, one sub-fertile bull would have a larger impact on the overall herd fertility than a single cow with fertility problems.

Establishing a successful pregnancy in dairy cattle is a complex process and there is strong evidence that service sire fertility has an important role. Relevant links between bull fertility and reproductive success have been observed not only in the fertilization process through differences in semen quality [5], but also in embryo preimplantation and development [6] as well as in offspring performance in later life [7]. Moreover, there is recent evidence that embryos from bulls with low fertility have a reduced ability to establish pregnancy and this depends on several factors such as sperm fertilizing ability, preimplantation embryonic development and development of the embryo and placenta after conceptus elongation and pregnancy recognition [8].

Genome wide association studies have been successful in using dense genetic markers such as single nucleotide polymorphism (SNP) markers to identify genomic regions affecting relevant phenotypes. Often, very stringent significant thresholds must be used in order to avoid multiple-testing problems, leading in general to lose both variants with small effect and rare variants [9]. In addition, these genome-wide dense markers have facilitated the so-called genome-enabled prediction, which aims to predict unobserved genetic values or yet-to-be observed phenotypes by regressing phenotypic values on SNP genotypes [10]. In animal breeding, genomic prediction is used to accurately select better animals at an early age as breeders for the next generation.

The accuracy of genomic prediction can be improved by either increasing the number of markers or the size of the reference population. The rationale behind increasing marker density or even using whole-genome sequence data is that the linkage disequilibrium between SNP makers and causative mutations is somehow maximized. However, incorporating high-density markers or even imputing SNP to sequence level may not result in higher prediction accuracies, as genomic prediction is affected not only by the reference population and the imputation process [11, 12], but also by the genetic architecture of the trait [13].

Generally, genomic prediction models assume that all markers in the genome have an effect in the trait of interest and that the marker effects have all the same magnitude [14]. However, there is evidence that different regions of the genome have different contributions to the genetic variability of a trait. For example, variants known to cause amino acid changes or variants within regulatory regions are commonly found among the most significant markers [15–17]. In the same manner, there are locations within a gene where variants are more likely to have a significant impact on the phenotype [18].

Functional annotation information can be used to prioritize groups of markers by assigning them different weights in the predictive models. Markers located near genes, affecting gene function or known to be causal mutations have been used to improve the accuracy of genomic predictions. A study by Wiggans et al. [19] reported a 1.4 percentage points gain across traits for Holstein cattle in the US national genomic evaluation by adding causative variants and removing less informative markers. MacLeaod et al. [20] proposed a modification to the BayesR method [21] called BayesRC, which incorporates prior biological knowledge about known genomic regions that are more likely to affect the trait of interest. BayesRC results showed that modeling these biological priors improved the accuracy of genomic prediction and also QTL discovery. In this sense, QTL markers identified by whole-genome scans can also be used to improve genomic prediction models [22, 23].

We recently reported promising results regarding the prediction of service sire fertility using 7.4 k US Holsteins bulls and 55 k SNP markers [24]. We concluded that the use of high-density SNP data together with the inclusion of functional information into the predictive models could improve the prediction of dairy bull fertility. As such, the first objective of this study was to assess the prediction of service sire fertility using the entire U.S. Holstein evaluation dataset and 300 k SNP markers across the genome. Previous studies from our group have shown that some genomic regions have marked effects on dairy sire fertility [25]. Therefore, the second objective was to evaluate the potential benefits of incorporating markers with large effect into genomic prediction models. Finally, the third objective of this study was to investigate the predictive power of genetic variants with relevant functional roles, such as non-synonymous, synonymous, or non-coding regulatory variants.

## Methods

### Phenotypic and genotypic data

The bull fertility phenotype evaluated in this study is sire conception rate (SCR), which represents the US national dairy bull fertility evaluation based on cow field data (pregnancy records). This is a phenotypic rather than a genetic evaluation because the fertility estimates include both genetic and non-genetic effects. The current model for SCR evaluation considers not only factors related to the sire under evaluation, but also factors, also known as nuisance variables, related to the cow that receives the unit of semen. These nuisance variables, such as cow age, parity and milk yield, could distort the measurements of bull fertility, and therefore, should be accounted for in the model [26, 27]. The variable SCR is defined as the expected difference in conception rate of a given bull compared with the mean of all the bulls evaluated.

A total of 11,539 Holstein sires with SCR records were used in this study. These SCR records belong to 29 consecutive evaluations released from August 2008 to April 2018. All records are freely available in the Council on Dairy Cattle Breeding (CDCB) website (https://www.uscdcb.com/) with the corresponding reliabilities calculated as a function of the number of breedings. Since there are sires with more than one SCR record from different evaluations, the most reliable SCR value, i.e. the record with most breedings was kept for the analyses. The Cooperative Dairy DNA Repository provided 312 k SNP data for all the 11,539 bulls with SCR records. SNP markers that either mapped to the sex chromosomes, presented a minor allele frequency below 5% or a call rate below 95% were removed. After quality control, a total of 295,159 SNP markers remained for subsequent analyses.

### Incorporating SNP with large effect and functional annotation data into genomic predictive models

**Fig. 1:**
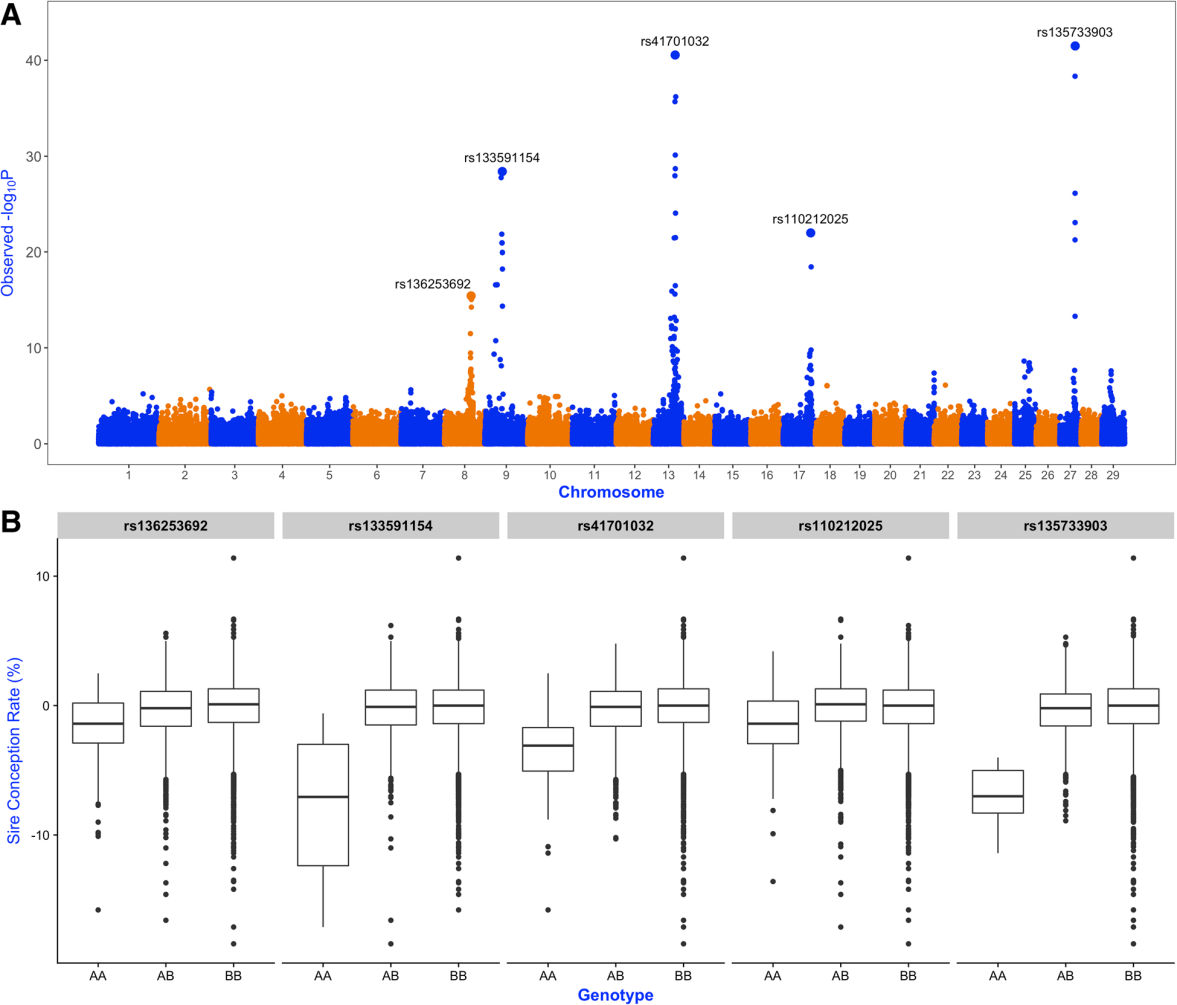
Whole-genome scan for dominance effects on Sire Conception Rate. **a** Manhattan plot showing the SNP significance across the bovine genome for dominance effects on Sire Conception Rate. **b** Boxplots showing the observed differences in Sire Conception Rate for each genotype of the five significant dominant markers

For the first objective, the predictive power of the entire high-density SNP dataset was evaluated using a whole-genome prediction model (**‘Base’** model), assuming that all markers have the same contribution to the phenotype. For the second objective, where the goal was to evaluate the benefits of including markers with large effect, dominance genetic effects were evaluated across the entire genome following the study by Nicolini et al. [25]. Briefly, each SNP was tested using a two-step mixed model-based approach. In the first step, the following mixed model, **y** = **Xb** + **Zu** + **e**, without including SNP was fitted. The random effects were assumed multivariate normal with $\mathbf{u}\sim N\left(0,\mathbf{G}{\upsigma}_{\mathrm{u}}^2\right)$ and $\mathbf{e}\sim N\left(0,\mathbf{I}{\upsigma}_{\mathrm{e}}^2\right)$. The variance–covariance matrix of this animal mixed model was estimated as ${\mathbf{V}}_0=\mathbf{ZG}{\mathbf{Z}}^{\prime }{\upsigma}_{\mathrm{u}}^2+\mathbf{I}{\upsigma}_{\mathrm{e}}^2$. In the second step, the following model was fitted for every SNP, **y** = **Xβ** + *X*_*SNP*_*β*_*SNP*_ + **ε**, assuming that $\boldsymbol{\upvarepsilon} \sim N\left(0,{\mathbf{V}}_0{\upsigma}_{\upvarepsilon}^2\right)$. For each X_SNP_, genotypes were coded as 0 for the AA and 1 for either AB or BB in order to test if a single copy of the B allele (reference allele) has the same effect on the phenotype as two copies. The significance of the SNP effect was tested using the following test statistic:

$$ \mathbf{z}=\frac{\mathbf{X}{\prime}_{\mathrm{SNP}}{\mathbf{V}}_0^{-1}\left(\mathbf{y}-\mathbf{X}\hat{\upbeta}\right)}{\sqrt{\mathbf{X}{\prime}_{\mathrm{SNP}}{\mathbf{V}}_0^{-1}{\mathbf{X}}_{\mathrm{SNP}}}} $$

which approximates the Wald test and is asymptotically standard normal. These analyses were performed using the *R* package MixABEL [28]. Genome-wide results were corrected for possible inflation of the test statistics using the function VIFGC implemented in the *R* package *GenABEL* [29]. The VIFGC function estimates corrected test statistics using a genomic control method based on the variance inflation factor. Results of this two-step mixed model-based approach revealed five SNPs with marked dominance effects (Fig. 1). These SNPs were then coded as 0 or 1, in order to represent the effect of having none or at least one copy of the B allele, and where fitted as fixed effects in an alternative whole-genome prediction models labeled as **‘Base + 5 SNP’** model, i.e., the base model plus five 0/1 markers fitted as fixed effects.

For the third objective, different SNP classes were evaluated based on the functional roles of the genetic variants. Gene annotations from the University of California Santa Cruz (UCSC) database (https://genome.ucsc.edu/) were downloaded and used to map and retrieve functional roles of the entire SNP dataset based on *Bos taurus* UMD3.1 genome assembly. Sequence Ontology terms are used in the UCSC database to describe the effect of each genetic variant on the structure of the gene transcripts. The SNPs were grouped into five functional classes (Table 1), namely *5’region*, *3’region*, *non-synonymous*, *synonymous*, and *ncRNA*. The classes *5’region* and *3’region* were defined as regions with possible regulatory effects. In the *5’region* class, we included SNPs located in the 5’UTR and within 5 kb upstream of the start codon of a gene. In the same manner, for the *3’region* class, we included SNPs in the 3’UTR or located within 5 kb downstream a gene. The class labeled as *non-synonymous* includes missense and nonsense genetic variants. Here, missense is defined as a sequence variant that changes one base and this change results in an alteration in the amino acidic sequence, while preserving the length of the final polypeptide. A nonsense sequence variant is defined as a change in one base that results in a premature stop codon leading to a shortened polypeptide. The SNP class *synonymous* represent those variants that change one base leading to a change in the codon, but with no resulting change to the encoded amino acid. Under *non-coding RNAs* (ncRNA) class are grouped genetic variants in the sequence of non-coding RNA genes, including tRNAs, ribosomal RNAs, and small RNAs. Finally, the set of SNPs that did not map to any of these five SNP classes were labeled as *intergenic*.

**Table 1:** Number of genetic markers mapped to different functional SNP classes

Class	Definition	Variant description	Number of SNP
5′ Region	Upstream gene variant	Located within 5000 bases of the 5′ of an annotated gene	7280
	5’UTR	Located in the 5′ untranslated region of an annotated gene	
3′ Region	Downstream gene variant	Located within 5000 bases of the 3′ of an annotated gene	4122
	3’UTR	Located in the 3′ untranslated region of an annotated gene	
Non-synonymous	Missense	Changes one base resulting in a different amino acid sequence, but the length of the polypeptide is preserved.	1144
	Nonsense	Changes one base resulting in a premature stop codon, leading to a shortened polypeptide.	
Synonymous	Synonymous	Changes one base but resulting in the same amino acid.	2090
ncRNA	ncRNA	Located in a non-coding RNA gene	1556

### Statistical models

In order to predict yet-to-be observed SCR values, different linear kernels (genomic matrices) were evaluated using Bayesian reproducing kernel Hilbert spaces regression models (RKHS) [30, 31]. Kernel-based regression procedures are powerful predictive machines that allow the incorporation of prior information about functional roles of markers using either single or multiple kernels.

#### Single-kernel model

Single-kernel models were fitted for either all the SNPs or each of the functional SNP subsets. Phenotypes were analyzed using the following model:

$$ \mathbf{y}=\mathbf{Xb}+\mathbf{K}\boldsymbol{\upalpha } +\mathbf{e} $$

where **y** is the vector of phenotypic records (SCR values); **b** is the vector of fixed effects including a general intercept (μ) and the SCR evaluation class effect; **X** is the design matrix relating fixed effects to SCR records; **K** is an *n × n* kernel matrix indexed by the SNP genotype matrix and takes the form **K** = **SS**^**T**^/*p*, where **S** is a matrix of centered and standardized SNP genotypes and *p* represents the number of SNPs, which is equivalent to the well-known additive genomic relationship matrix formulated by VanRaden [14] and Yang et al. [32]; **α** is the vector of RKHS regression coefficients estimated as the solution that minimizes *l*(**α**| λ) **=** (**y** − **Kα**)′(**y** − **Kα**) + λ**α**′**Kα**, where λ is the regularization parameter; and **e** is the error term. The random effects **α** and **e** were distributed as $\boldsymbol{\upalpha} \sim N\left(0,{\mathbf{K}}^{-1}{\sigma}_g^2\right)$ and $\mathbf{e}\sim N\left(0,{\mathbf{R}}^{-1}{\sigma}_e^2\right)$, where ${\sigma}_g^2$ and ${\sigma}_e^2$ are the genetic and residual variances, respectively, and **R** is an identity matrix.

#### Multi-kernel model

In this predictive model, multiple kernels were fitted simultaneously in order to evaluate the different functional SNP classes. The use of multiple kernels in one model allows to differentially weight one or more kernels that largely contribute to the trait of interest, overcoming at the same time possible loss in predictive ability due to the use of a single kernel [33]. Two alternative multi-kernel models, labeled as **‘Intergenic + Functional’** and **‘Intergenic + 5 SNP + Functional’**, were evaluated. These models included six different kernels representing the five functional SNP classes and the intergenic SNPs. These multi-kernel models were evaluated using the following equation:

$$ \mathbf{y}=\mathbf{Xb}+\sum \limits_{j=1}^i{\mathbf{K}}_j{\boldsymbol{\upalpha}}_j+\mathbf{e} $$

where *i* = 6 is the number of SNP classes and **K**_*j***,**_ with *j* = 1, 2, .., 6, is the linear kernel linking SCR records with each of the SNP classes. The random genomic and residual effects were assumed to be independent and normally distributed as ${\boldsymbol{\upalpha}}_j\sim N\left(0,{\mathbf{K}}_j^{-1}{\sigma}_{gj}^2\right)$, and $\mathbf{e}\sim N\left(0,{\mathbf{R}}^{-1}{\sigma}_e^2\right)$, respectively.

### Implementation of the analysis

All the RKHS models were run using Gibbs sampling. For each model, a Markov chain Monte Carlo (MCMC) with 100,000 samples (iterations) was run and the first 30,000 samples were discarded as burn-in. The remaining 70,000 samples were thinned at a rate of 5 resulting in 14,000 samples for computing features of the posterior distribution. Convergence of the chain was checked by visual inspection of trace plots of some key parameters, such as variance components. In addition, the converge of the multi-kernel models, arguably the most complex models used in this study, was also evaluated using Geweke. All these analyses were performed using the *R* package *Bayesian Generalized Linear Regression* (BGLR) [34].

### Model predictive ability

The predictive ability of the different RKHS regression models was assessed by 5-fold cross-validation. In this scenario, the entire data set (11,539 bulls with genotypes and phenotypes) was divided at random into five sets. Four out of the five subsets were combined to create the training population while the remaining subset was used as testing set. Phenotypes in the testing set where set to unknown and the training population was used to train the model in order to predict phenotypes for the testing set. Each of the five subsets was used as testing population one time. The entire five-fold cross-validation process was repeated ten times, therefore, each analysis resulted in 50 estimations. The predictive performance of each model was assessed using the Pearson product moment correlation (CORR) between observed phenotypes and predicted phenotypes in the testing population. Additionally, the mean-squared error of prediction (MSEP) was calculated as a measure of prediction bias and variability, using the following formula,

$$ \mathrm{MSEP}={n}^{-1}{\sum}_{f=1}^5\sum {\left(\boldsymbol{y}-{\widehat{\boldsymbol{y}}}_{test}\right)}^2 $$

where *n* is the number of animals in each fold (*f*), and *y* and $\widehat{y}$ are the observed and predicted SCR values, respectively. In order to evaluate the predictive ability of the different functional SNP classes, equal number of SNPs were randomly sampled from the entire genome creating a random set of SNPs for each functional SNP class. The random sampling was repeated 10 times, and the predictive ability of each set of random SNPs was assessed using the same five-fold cross-validation procedure described above. Therefore, for the random set of SNPs, each analysis resulted in a total of 500 estimates.

## Results and discussion

Service sire has an important role in establishing a successful pregnancy in dairy cattle. Semen from a single sire can be used to inseminate hundreds of cows, and therefore, the fertility of the service sire should not be overlooked. The accurate prediction of yet-to-be observed fertility phenotypes is very challenging, and the incorporation of different sources of information can help to improve model predictive performance. This study was specially conducted to investigate the feasibility of predicting dairy bull fertility using either markers with large effect and functional annotation data. We first evaluated model predictive ability using 300 k SNP markers and the entire U.S. Holsteins SCR dataset. Second, we investigated the impact of including non-additive markers with large effect on model predictive performance. Third, we assessed the predictive ability of different sets of functional SNP variants.

### Predicting ability of alternative whole-genome predictive models

**Fig. 2:**
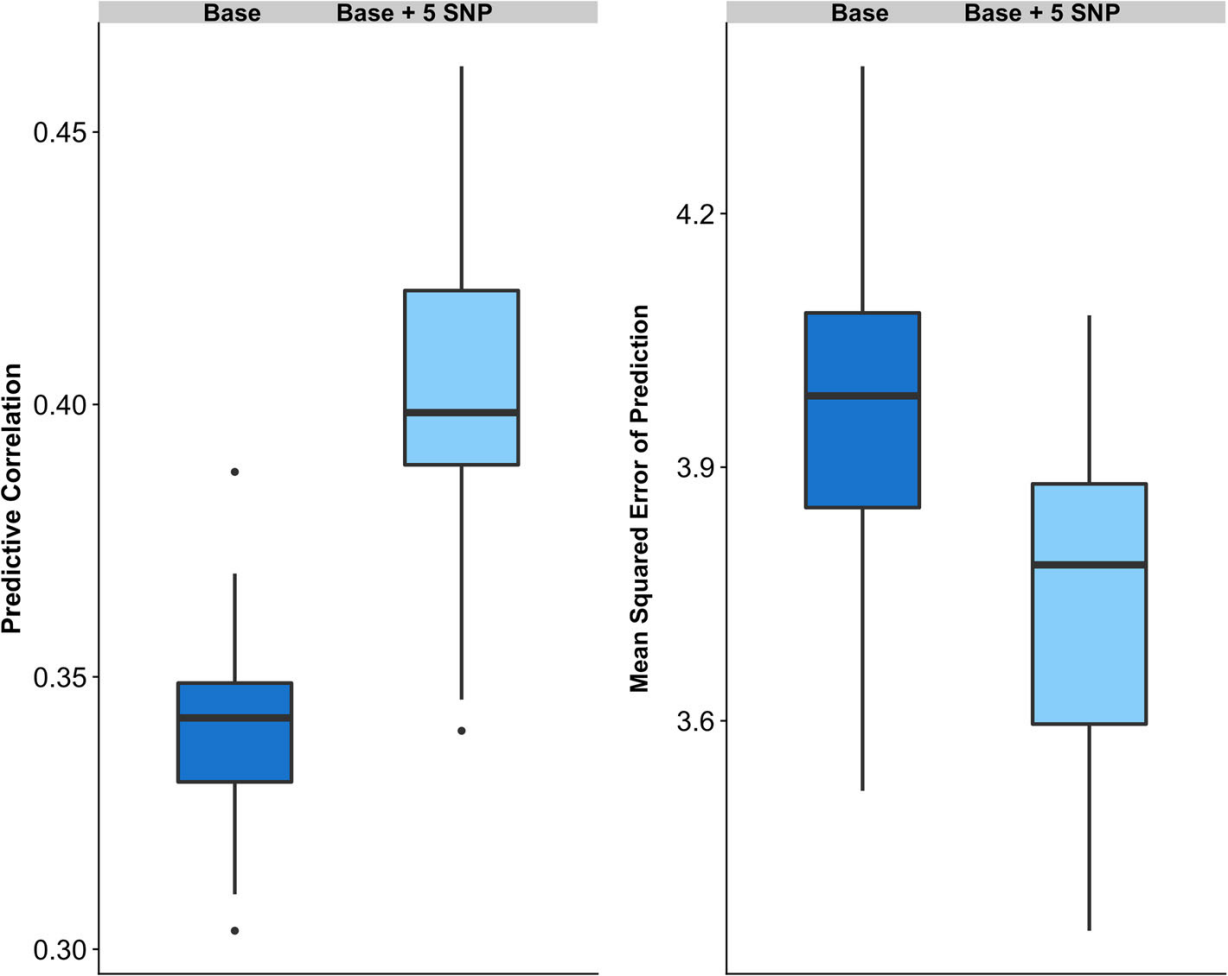
Predicting ability of alternative whole-genome predictive models. Predictive correlation (left) and mean squared error of prediction (right) was evaluated for each model. Blue boxes represent the ‘Base’ model that includes the whole SNP dataset (295,159 SNP). Light blue boxes represent the ‘Base + 5 SNP’ model that includes five non-additive SNPs fitted as fixed effects

Figure 2 shows the predictive ability of the **‘Base’** model using the entire SNP dataset in a single linear kernel, which is mathematically equivalent to the genomic-BLUP [14]. The predictive performance of this whole-genome model was contrasted with the predictive power of the **‘Base + 5 SNP’** model that includes five significant non-additive markers fitted as fixed effects. The **‘Base’** model exhibited an average correlation between observed and predicted SCR values of 0.340, and a mean-squared error of prediction equal to 3.973. The predictive ability of this model is in concordance with our previous study [24], where we used 54,807 SNPs and 7447 Holsteins bulls and reached an average CORR value equal to 0.341 and an average MSEP value equal to 4.160. Note that the MSEP value in this current study was lower, revealing that by increasing the size of the population (and therefore having a larger training population) and/or increasing the number of SNPs, prediction bias was reduced by 5%. Notably, model predictive ability was largely improved by including 5 markers with large effect. Indeed, **‘Base + 5 SNP’** model delivered CORR = 0.403 and MSEP = 3.761, representing an increase in predictive correlation of about 19% and a decrease in prediction bias by more than 5% compared with the standard model. The whole-genome scan for dominance effects showed that these five markers have a major impact on sire conception rate with extreme significance values (−log_10_*P-*values between 15 and 40; Fig. 1a). Each of these markers explain between 3 and 8% of the observed differences in conception rates between AB/BB and AA bulls (Fig. 1b). It is worth noting that these five markers presented negligible additive effects (data not shown). As reported by Nicolini et al. [25], these significant non-additive markers are near genes directly involved in male fertility, with functions closely related to testis development, spermatogenesis and sperm maturation. In consistency with our results, Lopes et al. [35] showed that including markers with relatively large effect improved model prediction ability for number of teats in 4 different pig populations. Similarly, Zhang et al. showed that the accuracy of genomic predictions can be improved by incorporating prior information into genomic models either from public QTL databases [36] or from the current dataset [37].

If we divide the average predictive correlation (0.403) by the square root of the heritability (*h*^2^ ≈ 0.30) we obtain a predictive accuracy equal to 0.735. This value is higher than the selection accuracies obtained for some health traits currently evaluated in U.S., such as ketosis and metritis, as well as higher than the accuracies reported for some calving ability traits, such as sire calving ease or sire stillbirth rate [38, 39]. Overall, our results provide further evidence of the importance of non-additive effects on dairy bull fertility, and the inclusion of these significant markers into the genomic predictive models markedly increases predictive ability and prediction accuracy.

### Predicting ability of different functional SNP classes

**Fig. 3:**
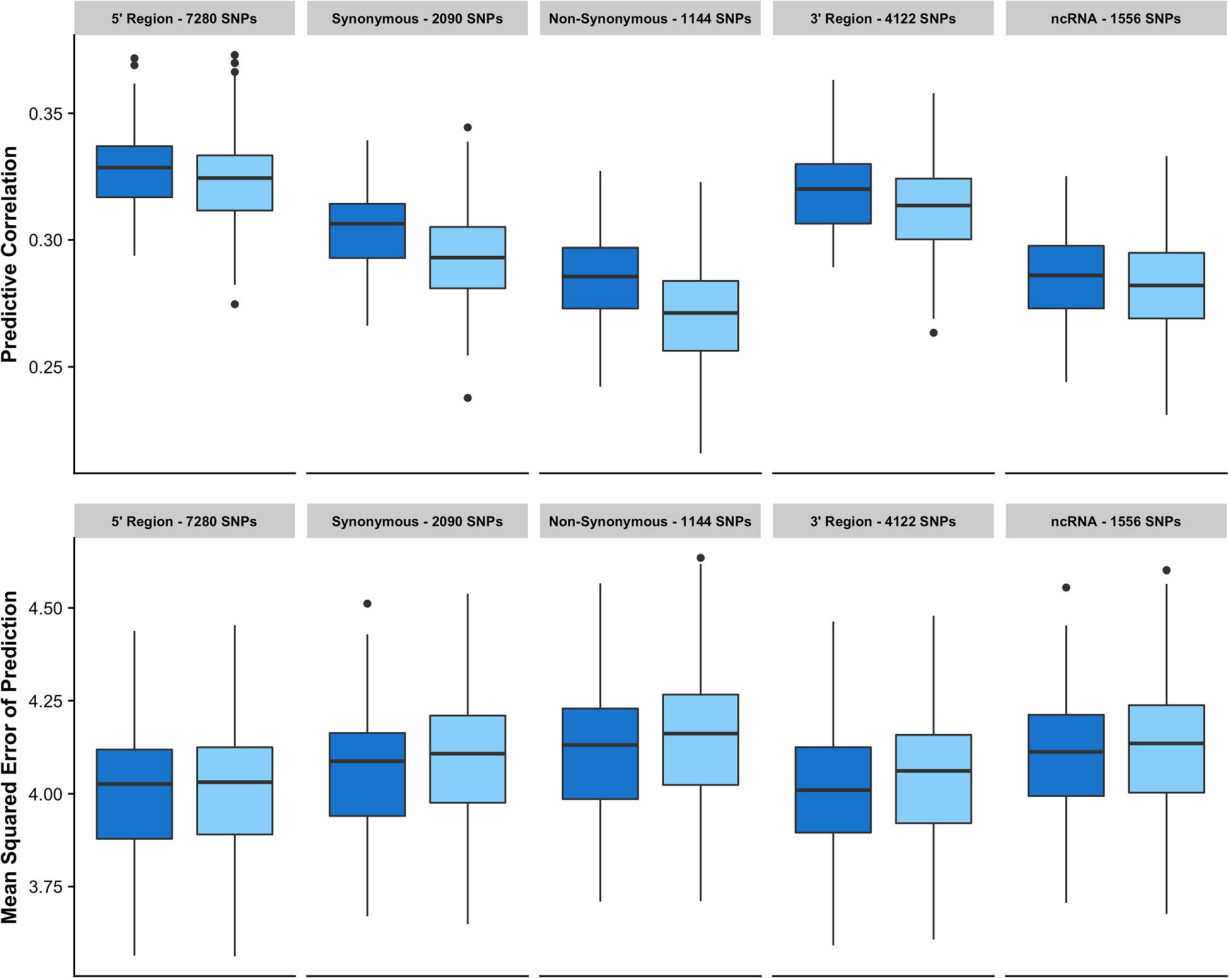
Predictive ability of different functional SNP subsets. Predictive correlation (top) and mean squared error of prediction (bottom) for each set of functional SNPs (blue), compared with the same number of SNPs but randomly sampled across the entire genome (light blue)

The prediction ability of each of the five functional SNP classes described in Table 1 was investigated using single-kernel models. Figure 3 shows the predictive correlation and the mean-squared error of prediction for each functional class, along with the corresponding results for the same number of SNPs but randomly sampled across the entire genome, i.e., random set of SNPs. Interestingly, the five functional SNP classes followed the same trend of higher CORR and lower MSEP than their counterparts using random SNPs. The class *non-synonymous* presented the largest difference in predictive correlation compared with random markers, 0.285 versus 0.271, representing an increase of about 5% in predictive ability. The SNP classes *synonymous* and *3’region* also showed sizeable differences in predictive correlation compared to randomly sampled SNPs, with increases between 4 and 2% in predictive ability. Koufariotis et al. [17] investigated the proportion of genetic variance explained by SNP classes for several traits in dairy cattle, and concluded that missense variants (included here in the *non-synonymous* class) and synonymous variants explained the highest proportion of variance compared with the rest of the SNP classes. Morota et al. [40] and Abdolahhi-Arpanahi et al. [41] also evaluated the predictive ability of different SNP classes in broiler chicken, and concluded that SNP in genic regions presented similar performance than those located in intergenic regions with a higher predictive correlation for the synonymous class. Despite the common belief that synonymous mutations have no major effects on the phenotype, in most genomes, synonymous codons have different frequencies, phenomenon known as “codon usage bias”, and there is growing evidence indicating that synonymous mutations are a source of natural variation, affecting both splicing and mRNA stability [42].

### Predicted ability of alternative multi-kernel models

**Fig. 4:**
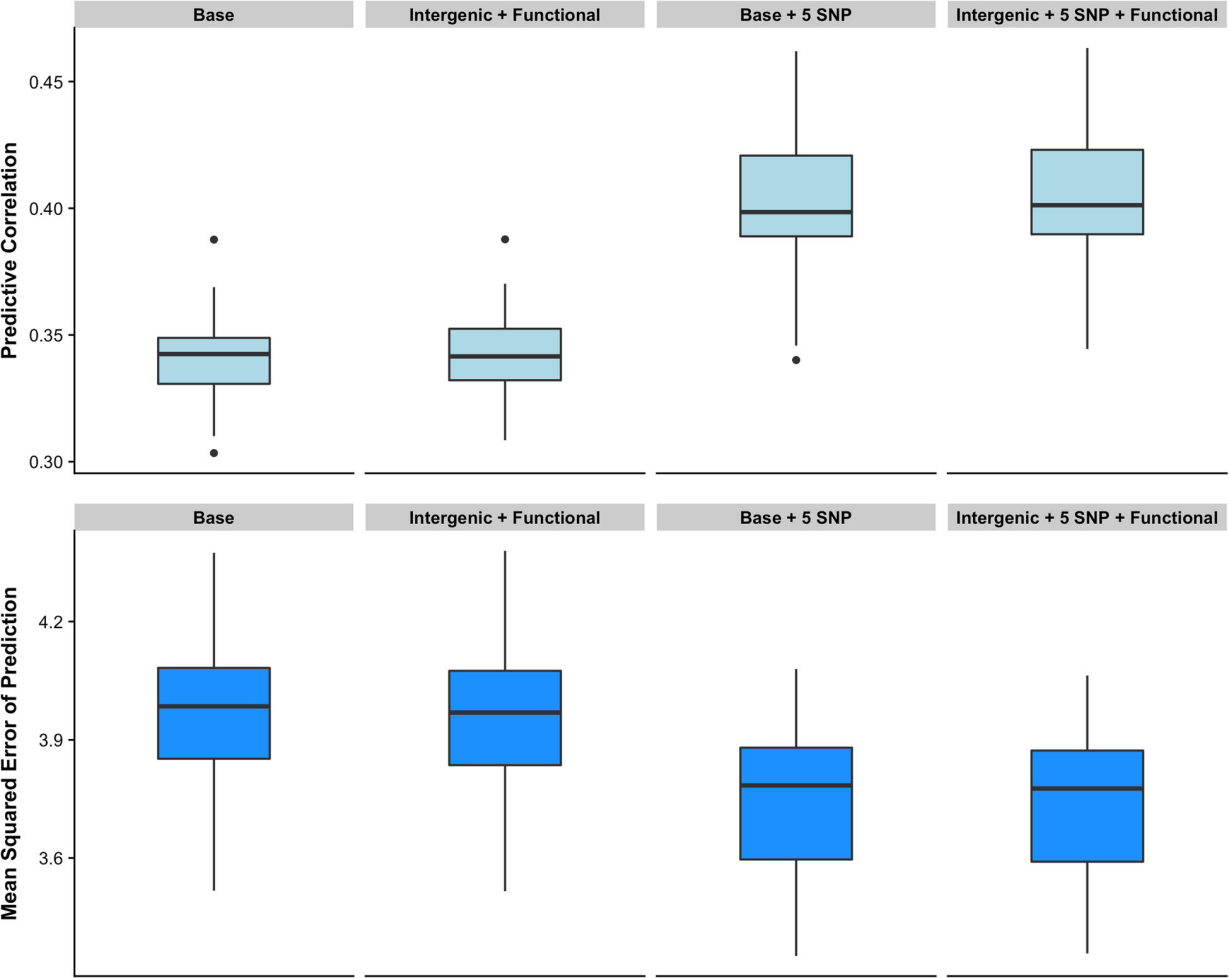
Predicting ability of alternative single-kernel and multi-kernel whole-genome predictive models. Predictive correlation (top) and mean squared error of prediction (bottom) for alternative single-kernel and multi-kernel whole-genome predictive models

Figure 4 shows the predictive performance of alternative multi-kernel models fitting the five functional SNP classes (functional) together with the intergenic SNPs. Note that the multi-kernel **‘Intergenic + Functional’** model (CORR = 0.342 and MSEP = 3.967) showed similar predictive ability than the single-kernel **‘Base’** model (CORR = 0.340 and MSEP = 3.973). Similarly, the multi-kernel **‘Intergenic + 5 SNP + Functional’** model (CORR = 0.405 and MSEP = 3.753) did not outperform the single-kernel **‘Base + 5 SNP’** model (CORR = 0.403 and MSEP = 3.761). Overall, each functional SNP class delivered higher predictive ability than its counterpart using random SNPs, however, multi-kernel models fitting all functional variants together did not outperform the standard whole-genome approach. It should be emphasized that in dairy cattle, linkage disequilibrium interferes with the use of biological information in prediction because irrelevant markers (SNPs without any biological role) capture part of the information encoded by relevant markers, causing that the intergenic and the functional SNP classes exhibit similar predictive abilities.

## Conclusions

The genomic prediction of service sire fertility is possible, and this could have a great impact on the dairy industry worldwide. Compared to our previous study [24], the inclusion of more animals in the training population and the use of more SNP markers did not improve predictive correlation but did reduce prediction bias. Results from the whole-genome scan confirmed the relevance of non-additive genetic effects in fitness-related traits, such as male fertility. Interestingly, the inclusion of five markers with large dominance effect into genomic predictive models markedly increased prediction performance. Moreover, the different functional SNP classes showed better predictive power than the randomly sampled SNP sets. These results indicate that the predictive power of these functional classes of SNPs is driven in part by their biological roles and not simply by accounting for population structure. However, multi-kernel models fitting functional annotation data showed similar predictive performance than the standard whole-genome approach. Overall, our findings emphasize the value of incorporating markers with large effect into prediction models. This is the foundation for the development of novel genomic strategies that can help the dairy industry make accurate genome-guided decisions, such as early culling of predicted subfertile bulls. Moreover, the inclusion of functional annotation data into genomic predictive models deserves further research. The use of whole-genome sequencing data plus a better annotation of the bovine genome might provide new opportunities in this field.

## Abbreviations

CORR: predictive correlation
MSEP: mean-squared error of prediction
SCR: sire conception rate
SNP: single nucleotide polymorphism
